# Finishing genomes with limited resources: lessons from an ensemble of microbial genomes

**DOI:** 10.1186/1471-2164-11-242

**Published:** 2010-04-16

**Authors:** Niranjan Nagarajan, Christopher Cook, MariaPia Di Bonaventura, Hong Ge, Allen Richards, Kimberly A Bishop-Lilly, Robert DeSalle, Timothy D Read, Mihai Pop

**Affiliations:** 1 Computational and Mathematical Biology, Genome Institute of Singapore 127726, Singapore; 2 Center for Bioinformatics and Computational Biology, University of Maryland, College Park, MD 20742, USA; 3 Naval Medical Research Center, 503 Robert Grant Avenue, Silver Spring, MD 20910, USA; 4 Henry M. Jackson Foundation, 1401 Rockville Pike, Rockville, MD 20852, USA; 5 American Museum of Natural History, New York, NY 10024, USA; 6 Division of Infectious Diseases & Department of Human Genetics, Emory University, Atlanta, GA 30322, USA

## Abstract

While new sequencing technologies have ushered in an era where microbial genomes can be easily sequenced, the goal of routinely producing high-quality draft and finished genomes in a cost-effective fashion has still remained elusive. Due to shorter read lengths and limitations in library construction protocols, shotgun sequencing and assembly based on these technologies often results in fragmented assemblies. Correspondingly, while draft assemblies can be obtained in days, finishing can take many months and hence the time and effort can only be justified for high-priority genomes and in large sequencing centers. In this work, we revisit this issue in light of our own experience in producing finished and nearly-finished genomes for a range of microbial species in a small-lab setting. These genomes were finished with surprisingly little investments in terms of time, computational effort and lab work, suggesting that the increased access to sequencing might also eventually lead to a greater proportion of finished genomes from small labs and genomics cores.

## Background

Not long ago, the expected outcome of a microbial genome project was the complete DNA sequence of all the chromosomes and extra-chromosomal elements of the genome being sequenced. As more and more complete genome sequences became available in public databases, scientists started to debate the need for completely sequencing the genome of an organism [1-3]. While an initial draft sequence for an organism could be determined in a matter of weeks, the complete sequence required many months or even years of additional experiments - a time- and cost-intensive process called genome finishing. Furthermore, draft assemblies are sufficient for many genomic analyses, especially if complete sequences of closely related organisms are available. The recent development of *nextgen *high-throughput sequencing technologies appears to have sealed the fate of genome finishing. Draft assemblies of approximately 5 bacterial genomes can now be generated in a matter of days (hours in fact, ignoring library preparation time) using a single 454 Titanium sequencing instrument. The costs associated with finishing, however, have not significantly decreased in recent years. The high costs of finishing experiments, thus, appear to only be justified for high-priority genomes.

Having finished or nearly-finished genomes is of course still a worthy goal as it enables a much richer set of genomic analysis. For example, the reliability of order-based genomic analysis such as studying operon structure and gene regulation as well as the granularity of comparative genomic studies are enhanced by the availability of finished genomes. In addition, the finishing process can substantially improve the quality of the data available to the community by identifying and fixing mis-assemblies and low-coverage regions. Fortunately, several characteristics of the new types of sequencing data, specifically increased depth of coverage and low representation biases in the sequencing libraries lend themselves well for finishing analysis. Draft assemblies can therefore be combined with additional sequence and map-based information to reduce the finishing effort. Here we describe our experience in doing this in the course of several finishing projects, highlighting the reduction in finishing effort as well as the feasibility of such projects in a small-lab setting. We also present the tools and approaches that were designed in our lab for this purpose (source code and executables available at http://cbcb.umd.edu/finishing). In combination with the *democratization of genomics *made possible by the reduced cost of sequencing, computational approaches such as the ones we describe here may help rectify the imbalance in the number of draft vs finished (or nearly finished) genomes that are available to the scientific community.

### Overview of finishing techniques

Prior to describing our results we briefly survey the main challenges encountered in finishing a genome and outline ways in which new technologies can be used to overcome these challenges. Detailed descriptions of these approaches will be provided in the methods section.

Finishing aims to overcome two major limitations of the shotgun sequencing process. First of all, the output of a genome assembler is generally fragmented due to difficulties in assembling repeat regions and to cloning/sequencing biases. Second of all, the assembled fragments frequently contain errors, either due to sequencing artifacts or to the incorrect reconstruction of repeats. The finishing process can thus be decomposed into two steps: gap closure, and assembly validation and refinement.

In gap closure, pairs of adjacent contigs are identified, then the genomic sequence spanning the gap between them is determined, traditionally through directed-PCR and primer-walking approaches. When mate-pair libraries are available, the adjacency of contigs can often be inferred from the mate-pair data and the gaps spanned by paired reads (sequencing gaps) can be closed relatively easily. Contigs whose adjacency cannot be inferred from mate-pair data, however, require expensive (and error-prone) combinatorial PCR experiments [4]. New experimental technologies alleviate these difficulties in two ways. First of all, in nextgen sequencing projects performed to a high depth of coverage (>20-fold is common in 454 projects) sequence gaps between contigs are rare due to the relatively unbiased libraries generated by these new technologies; fragmentation into contigs is largely due to the presence of repeats. Therefore, often, once the adjacency between two contigs is determined (e.g. through PCR experiments), the contigs can be simply "glued" together without the need for additional sequencing. Second of all, the adjacency of contigs can be easily determined either through recently developed nextgen mate-pair protocols or through the use of new mapping technologies, such as the optical mapping approach from Opgen Inc. http://www.opgen.com.

The validation and refinement finishing stage aims to correct errors in the assembled sequence - both single-base errors (such as mis-called bases due to sequencing errors) as well as large-scale errors (such as mis-assemblies due to repeats). Both problems are somewhat alleviated in nextgen sequencing data. Due to a high level of coverage, most single base errors can be automatically corrected. This is true even in the case of 454 pyrosequencing where errors within homo-polymer tracts are common. Furthermore, assembly software designed specifically for high-coverage nextgen data (e.g. the Newbler assembler from 454) use conservative algorithms specifically designed to avoid mis-assemblies. The resulting assemblies are usually more fragmented; contigs end at repeat boundaries where the reconstruction of the genome is ambiguous. The high depth of coverage and conservative assembly strategy also enable a better estimation of the number of repeat copies contained within a contig. Repeat-induced ambiguities can be resolved through targeted PCR experiments aimed at uncovering the correct adjacency of the assembled contigs. As in the case of gap closure, once two contigs have been determined to be adjacent in the assembly, they can be simply glued together. In the following section we describe the results from our experience in putting these principles into practice.

## Results and Discussion

Throughout the following paragraphs we describe several genome projects that represent a range of sequencing strategies and genome characteristics. These genomes were sequenced through older versions of 454 pyrosequencing technology where read lengths were in the 100-200 bp range; our results should therefore also be valuable for newer projects with Solexa and SOLiD sequencing. The genomes we describe are: *Aggregatibacter aphrophilus *- sequenced through a hybrid 454-Sanger approach, several *Yersinia *species - sequenced through 454 pyrosequencing and augmented with optical mapping data and *Rickettsia prowazekii *- sequenced through 454 pyrosequencing and finished entirely through *in silico *methods. In all these cases, the finishing effort involved only tens of PCR reactions and finishing reads as opposed to the hundreds and even thousands that were required in some contemporary projects (see Table 1).

**Table 1:** Statistics from some contemporary finishing projects.

Genome	Size (Mbp)	Sequencing Center	Release Date	Finishing Reads
*Yersinia pestis *Angola	4.68	TIGR	12/12/07	5,642
*Frankia sp*. CcI3	5.4	JGI	02/06/06	2,417
*Vibrio cholerae *O395	4.1	TIGR	05/08/07	4,521
*Salmonella enterica *SL476	4.99	JCVI	07/24/08	799
*Pantoea stewartii *stewartii	-	Baylor	Ongoing	524
*Verrucomicrobium spinosum *DSM4136	8.2	JCVI	Ongoing	3,828
*Aliivibrio salmonicida *LFI1238	4.6	Sanger	10/01/08	2,033

Data was collected from NCBI's Trace Archive and Genomes Database. The *V. cholerae *genome was sequenced using a 454/Sanger hybrid approach while the rest of the genomes were sequenced by Sanger sequencing. Note that the *P. stewartii *genome was found to be particularly hard to finish, despite the high sequence coverage using Sanger sequencing, because of the presence of numerous plasmids in the sequenced strain.

### Finished genome of *A. aphrophilus*

The *A. aphrophilus *genome project was started at the American Museum of Natural History in 2007 to better understand the pathobiology of the bacterium [5] (GenBank accession number CP001607). *A. aphrophilus *is a proteobacteria from the Pasteurellaceae family that is strongly implicated as a causative agent of infective endocarditis [6]. It can also be found as an apparently benign resident of dental plaque.

Sequencing was done for this project using a hybrid approach, combining two runs of 454 pyrosequencing with Sanger sequencing of two shotgun libraries of clones of 1.6 to 4 Kbp inserts. Overall, 549, 417 pyrosequencing reads (average length ~100 bp) and 12, 889 Sanger reads (average length ~600 bp) were used to generate 22× and 3× coverage respectively of the 2.3 Mbp genome. The hybrid-assembly of these reads was constructed using a novel pipeline (see Methods) and with a Newbler assembly of the 454 reads as a starting point. In terms of sequence information, the Sanger reads added very little (≤0.2%) to the Newbler assembly (only 7 reads were not mapped to the Newbler assembly). However they provide valuable information to merge contigs (reducing contig count from 146 to 122 and increasing N50 size from 99 Kbp to 150 Kbp) and scaffold them (resulting in 110 scaffolds with an N50 size of 232 Kbp), as well as provide indpendent verification of the assembly (266 reads spanned across Newbler contigs and 246 mates connected the contigs).

**Figure 1:**
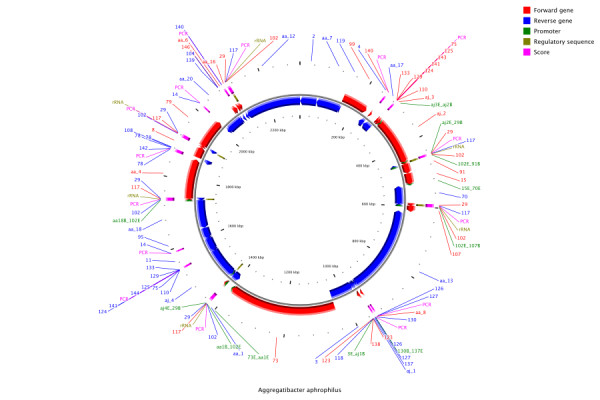
**Summary of the finishing effort for *A. aphrophilus***. As can be seen from the figure much of the finishing effort (PCR experiments indicated by bars in the outermost ring) were devoted to disambiguating the neighborhood of the rRNA operon.

After assembly and scaffolding, the *A. aphrophilus *genome was largely in 18 non-repeat scaffolds and 6 repeat scaffolds (one of these being a 6-copy scaffold containing the rRNA operon) - a very good starting point for our finishing efforts. The main effort of our finishing work was devoted to disambiguating repeats, particularly the rRNA operon. A schematic representation of these efforts can be seen in Figure 1. In total, only 42 PCR amplicons and 17 sequencing reactions were needed for completion, a task that is well within the scope of a small lab.

### Scaffolded assemblies of 8 *Yersinia *strains

The *Yersinia *genus contains several enteropathogens such as *Y. enterocolictica *and *Y pseudotuberculosis *and the causative agent of the plague *Y. pestis*. The genus also contains several less virulent strains that are commonly found in soil and water. While the genomes of multiple strains of the pathogenic *Yersinia *are available we know little about their non-pathogenic relatives and so correspondingly 8 other species *Y. kristensenii*, *Y. aldovae*, *Y. mollaretii*, *Y. fredriksenii*, *Y. bercovieri*, *Y. intermedia*, *Y. rohdei *and *Y. ruckeri *were sequenced in 2006 and 2007 via 454 FLX sequencing at the Naval Medical Research Center (GenBank accession numbers ACCA00000000, ACCB00000000, AALD00000000, AALE00000000, AALC00000000, AALF00000000, ACCD00000000 and ACCC00000000 respectively) [7].

The reads for the 8 species were assembled using the Newbler assembler and a summary of the sequencing and assembly statistics for the *Yersinia *genomes is given in Table 2. The assembly of these genomes was fragmented into many contigs and correspondingly was not suitable for comparative analysis or finishing of the genomes. In order to scaffold the contigs, optical restriction maps were generated for all the genomes (using the enzyme AII) and in many cases two maps were generated (using the enzyme AflII and NheI). The statistics for these maps are summarized in Table 2.

**Table 2:** Assembly and Map statistics for the *Yersinia *genomes.

	454 Contigs			Optical Map		
**Strain**	**Coverage**	**Count (>10 Kbp)**	**N50 Size (Kbp)**	**Size (Mbp)**	**Fragments**	**N50 Size (Kbp)**

*Y. kristensenii*	29×	488 (50)	93	AflII: 4.63	350	5.5

*Y. aldovae*	25×	375 (83)	86	AflII: 4.30	360	11.4

*Y. mollaretii*	36×	1637 (74)	85	AflII: 4.93	397	19.0

				NheI: 4.92	556	6.4

*Y. fredriksenii*	29×	1244 (46)	150	AflII: 5.34	467	24.7

				NheI: 5.39	611	3.6

*Y. bercovieri*	26×	1219 (84)	59	AflII: 4.54	415	2.6

				NheI: 4.50	591	13.9

*Y. intermedia*	35×	1242 (60)	124	AflII: 4.95	436	6.6

				NheI: 5.06	537	10.2

*Y. rohdei*	22×	281 (59)	116	AflII: 4.65	413	11.3

				NheI: 4.64	458	12.7

*Y. ruckerii*	36×	419 (63)	79	AflII: 3.90	142	29.6

				NheI: 3.95	457	7.9

For the 454 contigs we report the average coverage of the contigs, the number of contigs (with large contigs in parentheses) and the N50 size. For the optical maps, we report the total size, number of fragments and N50 size of fragments for AflII and NheI based maps on seperate lines.

The contigs were scaffolded on the optical maps using the SOMA package [8] (see Methods). As can be seen in Table 3, the resulting scaffolds contain a large fraction of the sequence in the genome, with few large gaps in the map. This is particularly the case for genomes scaffolded with multiple optical maps and where few large islands contain no restriction sites. It should be noted that unlike scaffolds obtained with mate-pairs, the scaffolds here are *genome-wide *and one per genome and therefore well-suited for finishing efforts.

**Table 3:** Scaffolding results for the *Yersinia *genomes.

Strain	AflII based	NheI based	Both Maps	Draft genome	
	Size in Mbp (% of genome)	Size in Mbp (% of genome)	Size in Mbp (% of genome)	Size in Mbp (% of genome)	# of gaps (>10 *Kbp*)
*Y. kristensenii*	3.91 (83.9)			4.36 (93.7)	37 (8)
*Y. aldovae*	3.51 (82.9)			3.64 (86.2)	39 (14)
*Y. mollaretii*	3.73 (76.1)	3.95 (80.6)	4.15 (84.8)	4.30 (87.8)	56 (24)
*Y. fredriksenii*	4.34 (81.0)	4.54 (84.8)	4.63 (86.3)	4.72 (88.1)	33 (20)
*Y. bercovieri*	3.32 (72.8)	3.50 (76.7)	3.62 (79.2)	3.87 (84.8)	57 (25)
*Y. intermedia*	4.38 (86.9)	4.14 (82.0)	4.35 (86.3)	4.62 (91.5)	53 (15)
*Y. rohdei*	3.91 (85.3)	3.81 (82.9)	4.01 (87.3)	4.15 (90.4)	40 (16)
*Y. ruckerii*	1.93 (49.4)	3.13 (80.1)	3.20 (82.0)	3.34 (85.5)	39 (17)

Here we report the size of the scaffolds obtained by combining each of the optical maps and the 454 contigs. These scaffolds were then merged and augmented with contig graph information (see Methods) to obtain the draft genome and we report the results after both stages. For the draft genome we also report the number of gaps in the final scaffold.

To further augment and verify the scaffolds, information about adjacency of contigs was extracted from the assembly and applied (see Methods). The results from this process can be seen in columns 5-6 in Table 3. In all cases, the placements from the optical map were confirmed and in a few cases gaps were closed *in silico*. The resulting draft genomes were well-suited for comparative analysis and as a template for finishing efforts; as a proof-of-concept project we worked on the *Y. rohdei *genome using a suite of finishing techniques (see Methods). Using only 43 PCR experiments and 26 sequencing reactions 33 of the gaps were closed, leaving only 7 gaps to close. In contrast, working with the original assembly (59 large contigs) could have necessiated on the order of 59^2 ^≈ 3000 PCR experiments (see Table 1). A similar project for the *Y. ruckeri *genome is also in progress.

### *In-silico *finishing of *R. prowazekii*

The *R. prowazekii *genome is an interesting case where we were able to finish the genome without any additional experimental effort. We believe it is the first case to be reported in the literature of the *in silico *closure of a bacterial genome and in fact we obtained similar results with several other Rickettsial genomes (data not shown).

**Figure 2:**
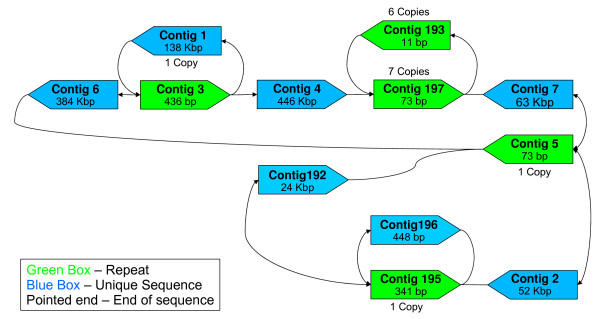
***R. prowazekii *Contig Graph**. Note that the comments for Figure 3 are also valid here. This graph can be resolved into a unique *in silico *reconstruction of the genome.

*R. prowazekii *is a Gram negative, aerobic bacterium that is the causative agent of epidemic typhus. In order to understand the genetic basis for phenotypic variation between various laboratory strains, a strain of *R. prowazekii *Madrid E was sequenced using a single run of a 454 FLX instrument to more than 100× coverage. The reads were assembled using Newbler into 197 contigs with an N50 size of 450 Kbp. Further analysis of the contig adjacency information however revealed that the entire circular genome can be reconstructed into a single gap-free sequence *in silico *(see Methods and Figure 2). Note that the fragmentation of the genome was not an assembler issue - assemblies using Euler-SR [9] and Celera Assembler [10] were in fact slightly worse (>260 contigs).

The *R. prowazekii *genome provides evidence for the power of high-coverage sequencing using new sequencing technologies such as 454; we were able to reconstruct the genome with no gaps using a single run of the 454 machine, *de novo *assembly using Newbler and analysis of the resulting contig adjacency information. Also, while the assembly was performed without using the prior knowledge of the previously sequenced *R. prowazekii *Madrid E genome [11], the assembly could be validated using it. The post-assembly analysis revealed that the two genomes align perfectly (indicating that there are no misordered contigs), differ in length by only 3 bp and are more than 99.96% identical in sequence.

## Conclusions

It should be noted that the finishing projects presented here were not selected in any way for their ease of finishing. While we of course cannot claim to have done a uniform sampling of "typical" genomes, the evidence from our experience as well as similar anecdotes from other labs, strongly suggests that it is increasingly feasible for small labs and genomic cores to take on the challenge of finishing a genome using minimal resources (possibly a week's effort for a bioinformatician and a lab technician).

As sequencing technologies improve in terms of their read lengths and throughput, the task of finishing a genome is likely to become even more feasible. In particular, technologies such as Illumina and SOLiD are already or will soon produce reads as long as some of those studied here (~100 bp) making the techniques described here directly applicable to the resulting assemblies. Also, newer 454 instruments, where read lengths are now roughly 400 bp, allow for the extension of similar ideas to larger genomes. As techniques to construct ordered restriction maps (such as nanocode maps [12] and nano-fluidic arrays http://www.bionanomatrix.com/) and mate-pair libraries improve, scaffolding and finishing larger genomes could potentially become a routine affair. All of this portends well for a more widespread adoption of finishing as a goal for genome projects.

Computational analysis continues to play an important role in the finishing task and it is unlikely that there will be a one-size fits all solution or a standard pipeline for this problem. However, often a small bag of tricks can make the task less daunting and an increased awareness and availability of tools for these could go a long way in making more finished genomes available to the scientific community. One such trick is the use of optical maps to aid in finishing and the results reported here represent the first large scale, automated use of these maps for scaffolding genomes. A slightly less novel and yet neglected idea is the contig adjacency information residing in assemblies (discussed originally in [13]). Ignoring it would have led us to potentially do hundreds of PCR experiments to determine the order of contigs in the *R. prowazekii *genome; instead we closed the genome *in silico*. As genomes are sequenced with longer reads and to greater coverage, we are likely to come across more such examples, possibly with larger, but still resolvable contig graphs.

The programs and pipelines that we used for our finishing analysis are freely available at http://cbcb.umd.edu/finishing. We hope that they can serve as a starting point as other labs strive to create and refine their own finishing toolboxes.

## Methods

In the following paragraphs we briefly describe some of the ideas and approaches that were used in the finishing projects described in this paper. More details about installation and running of the associated tools can be found in the documentation accompanying the tools.

### Using mate-pairs and contig adjacency information

Assembly algorithms piece together reads into contigs, which are ungapped sequences from the genome [14]. If additional information linking these contigs is available (such as mate-pairs) then they may also produce scaffolds, which represent regions of the genome where the contiguous sequence is not necessarily known, but the region can be represented as an ordered and oriented list of contigs with estimates for the lengths of the gaps between contigs.

**Figure 3:**
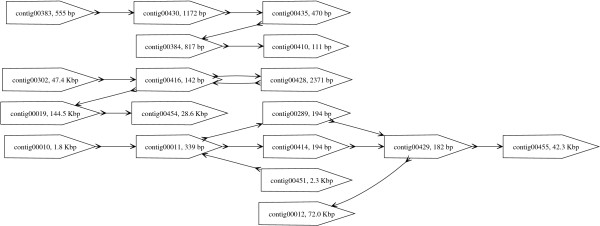
**Partial Contig Graph of *Y. kristensenii***. The pointed boxes represent contigs while the edges mark the presence of reads that span the corresponding contigs. The arrows on both ends of an edge indicate the orientation of the adjacent contigs. An arrow "out" of a contig indicates that the end of the contig is adjacent and an arrow "in" indicates that the beginning of the contig is adjacent.

Due to short read lengths, typical assemblies of reads from new sequencing technologies are fragmented into many small contigs. In many cases, cost, efficiency and technological hurdles exist for obtaining mated-reads. However, even in the absence of linking information, the contigs obtained from an assembler are not necessarily independent sequences. The contigs, in fact, are linked together, a structure that we refer to as contig graph, and this information can be extracted from the output of many assemblers (such as Newbler (454 Life Sciences), Celera Assembler [10] and Minimus [15]). An example of such a graph can be seen in Figure 3, obtained from the Newbler assembly for *Y. kristensenii*. The edges in such a graph arise from reads that span the corresponding contig ends.

The existence of information about the adjacency of contigs in an assembly is not a new concept. Surprisingly, however, it is common practice for finishing-associated tools to treat contigs from an assembly as sequences whose relative order and orientation is completely unconstrained [16,17]. In our work we found contig-graph information useful in three different tasks: 1) Closing gaps *in silico *as described in the section after next 2) Guiding and verifying map-based or mate-pair based scaffolding of contigs (see below and the next section) and 3) direct reconstruction of larger contigs. For the third task we relied on the fact that often these graphs have linear paths or simple repeat structures that can be easily resolved to merge contigs together (see Figure 3) and this is implemented as part of the AMOS-Hybrid pipeline described below. More sophisticated analysis as suggested in [18] to identify regions in the assembly or contig graph that are uniquely traversable is also feasible. In the case of the *R. prowazekii *genome, we reconstructed the contig graph using *get_graph.pl *in the finishing scripts package (see Availability) and manually noted the presence of a unique directed traversal of the graph to reconstruct the genome. Tools automating this task are still in development.

**Figure 4:**
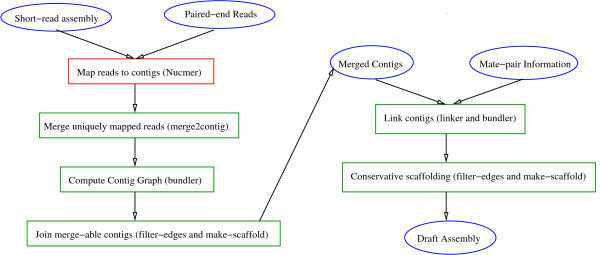
**AMOS-Hybrid pipeline**. Circles are used to represent input/output and intermediate datasets. Names in parentheses refer to the programs used to perform the corresponding tasks in the boxes.

In addition to contig graphs, where available, mate pair information can be invaluable to order and orient contigs and simplify the finishing task. With the availability of second generation sequencing technologies, several assemblers allow combinations such as 454 and Sanger mated reads (e.g. Celera Assembler [19], Newbler) or 454 and Illumina reads (e.g. VCAKE [20], MIRA [21]). Fewer assemblers, however, allow arbitrary combinations. In early 2007, such *hybrid *assemblers were unavailable and consequently the *A. aphrophilus *genome was assembled using a simple pipeline based on the AMOS package http://sourceforge.net/apps/mediawiki/amos. This pipeline allows the user to merge mated-reads from any sequencing technology into an existing assembly to scaffold and refine it (see Figure 4).

### Optical map based scaffolds

**Figure 5:**
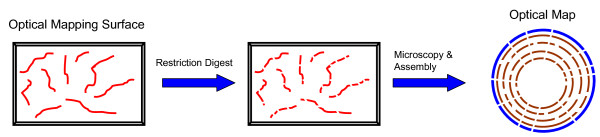
**The optical mapping process**. To generate a whole-genome optical map, DNA is sheared into fragments that are stretched and fixed onto an optical mapping surface and then digested using a restriction enzyme. The resulting pieces are optically analyzed and assembled into a genome-wide map.

While paired-end reads can be invaluable to scaffold contigs, they provide local order information and using them to recreate a genomewide ordering of contigs is computationally challenging. In addition, for time-critical applications in a biodefense or clinical setting, the time to construct paired-end libraries can be a limiting factor. In such settings, Optical Restriction Mapping [22], a form of ordered restriction maps (see Figure 5), can be a promising alternative as it can quickly provide genomewide restriction site information that can be used to order and orient contigs [8]. In particular, we used the SOMA package freely available at http://www.cbcb.umd.edu/soma to construct a genome-wide scaffold for assembled contigs using the information in corresponding optical maps (obtained from Opgen Inc). From a finishing perspective, these scaffolds are particularly useful, as for a set of *n *contigs, they help reduce the number of PCR experiments needed from roughly *n*^2 ^to *n *[23].

In the case of some genome projects, multiple optical-maps can be useful as they can provide complementary information and help place contigs that have few restriction sites. In addition multiple maps can also help validate placements from individual maps. In order to achieve this we implemented an efficient approach to merge single map results from SOMA and an overview of this approach is provided below:

Input = A set of contig placements on optical maps.

1. Select a contig placed on all the maps as an *anchor*.

2. For all uniquely placed contigs, test if the distance to the anchor is consistent across maps.

3. For non-uniquely placed contigs, find a unique placement based on agreement between maps.

4. Starting from the closest to the anchor, add unique placements into a global placement, excluding those that conflict with earlier placements.

The optical map based scaffolds can also be augmented with the information available in contig graphs and we employed it in two ways: 1) to validate placements using the optical map 2) to place smaller contigs based on connections to contigs already placed on the map. We found both these approaches useful in increasing the completeness and reliability of our contigs and the scripts we used are freely available (see Availability).

### Directed finishing using contig composition and adjacency information

Closing of gaps between contigs using directed finishing experiments can be a long and laborious component of a sequencing project. While map and mate-pair based scaffolding can be critical to reducing the number of experiments to be done, they will not typically eliminate them. It is therefore important in such cases to use auxilliary information to prioritize experiments. One approach that proved valuable in our efforts was to use contig adjacency information to resolve repeats, as in the case of the *R. prowazekii *genome (based on the unique traversal property discussed in Theorem 7.5 in [24]). In several cases, analysis of connecting paths in contig graphs helped close gaps *in silico *and the scripts we used for this are freely available (see Availability). Another useful strategy, particularly for the larger gaps, was to use sequence composition of contig ends to suggest contigs that could supply the missing sequence. In the case of *Y. rohdei*, for example, we matched GC content for 1 Kbp ends of contigs and the top match closed 5 large gaps (as validated by PCR experiments) using 5 of the 6 large contigs (>10 Kbp) that were not placed on the scaffold. A script to do this matching is also provided. Note that another similar approach that could prove valuable for closing gaps in future projects is to identify genes fragmented into two or more contigs to identify contigs that are likely to be adjacent [25].

## Availability

The executables and source code for the various programs used are avaialble at http://cbcb.umd.edu/finishing. This includes programs and scripts for scaffolding using optical maps (SOMA v2.0), an AMOS pipeline for merging mate-pair and contig information (AMOS-Hybrid v1.0) and scripts for various finishing tasks (Finishing Scripts v1.0).

## Authors' contributions

MB and RD designed the *A. aphrophilus *study, did the sequencing and the finishing experiments. HG and AR sequenced the *R. prowazekii *strain. CC, KB and TR designed the *Yersinia *study, did the sequencing and the finishing experiments. NN and MP did the computational analysis and drafted the manuscript. All authors read and approved the final manuscript.
